# Keeping Tabs on the Women: Life Scientists in Europe

**DOI:** 10.1371/journal.pbio.0040097

**Published:** 2006-04-11

**Authors:** Karla M Neugebauer

## Abstract

To increase the visibility of European women from post-docs to senior group leaders, the European Life Science Organization (ELSO) has created a Database of Expert Women in the Molecular Life Sciences.

The proportion of leading women in science is so small that it can be difficult to know who they are. To increase the visibility of European women from post-docs to senior group leaders, a Database of Expert Women in the Molecular Life Sciences ( http://www.elso-cdc.org) has been created by the European Life Sciences Organization (ELSO). The database is unique because it is designed for professional scientists to use: each entry in the database highlights publications in international journals, and familiar keywords and career stages. The goal is to provide a tool to help the scientific community work toward gender equality in Europe.

A simple definition of gender equality is that individuals, regardless of their sex, have equal chances of succeeding. For decades now, approximately half of the graduate students in the molecular life sciences in Europe and the US have been women. Given that these students are selected for their academic achievements and potential to perform as scientists, clearly selection committees and PhD supervisors believe that men and women are equal in their intellectual and research capabilities. Nevertheless, plotting the percentage of women holding pre-doctoral, post-doctoral, junior group leader, and professor positions shows a dramatic and steady decline for women as career stages advance, though numbers differ by country and scientific discipline [ 1, 2]. In the UK (1996–97), for example, ∼52% of postgraduate students in the biological sciences were women, compared to only ∼6% of professors [ 1]. Recent statistics for Germany are similar ( Figure 1). Because the corresponding curve for men intersects with the line for women, this generates the familiar “scissors diagram.” In 2001, women held an average of 8.9% of senior academic research positions in 17 EU and associated countries [ 1]. These data describe an existing gender inequality.

**Figure 1:**
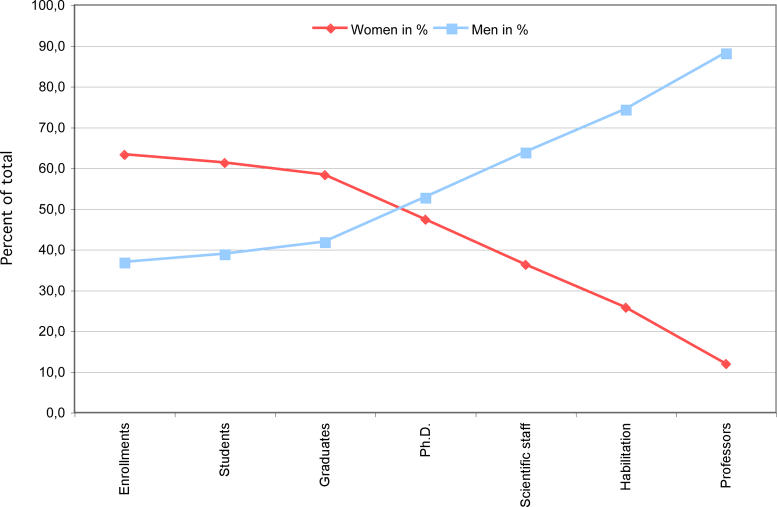
Scissors Diagram Showing the Gender Distribution within Career Stages in Biological Sciences at German Universities (2003) Percent male and female are shown for enrolling university students through graduation, PhDs awarded, scientific staff (includes post-doctoral fellows, some junior group leaders, and research scientists with university or PhD degrees), Habilitation (awarded for research accomplishments following the PhD as well as teaching experience, often a pre-requisite for university professorships in Germany), and professors (includes independent group leaders roughly equivalent to assistant, associate, and full professors in the American system—C2–C4 scale). Data prepared by Center of Excellence Women and Science (CEWS, http://www.cews.org/cews/index.php). Source: Federal Statistical Office Germany.

Won't the pool of women training today drive us naturally toward gender equality as time goes by? Historically, the answer is no. A study of cohorts of German scientists followed over time has shown that the size of the female pool entering scientific fields does not result in proportional achievement of senior positions [ 3]. Governments and scientific organizations are logically concerned about the failure of women to progress in science because they provide the resources for scientific education and training—from primary schooling to university education, to pre- and post-doctoral fellowships.

Their gamble is that this investment will provide returns in the form of discovery and technological innovation. If 50% of the beneficiaries do not advance within their fields, this is perceived as a waste of education and training. Clearly, not every post-graduate student of science can become a professor—there are simply not enough professorships to go around. However, we place faith in our merit-based system of hiring and funding as the means of selecting the best talent to lead science, technology, society, and our economies into the future. But unless something changes, much of our female talent will continue to be permanently lost to science.

Zena Werb, president of the American Society for Cell Biology (ASCB), recently observed that “we are seeing a trend in which women scientists seek, take, or are offered less challenging positions, as well as a continuing trend in which women rise through the ranks less efficiently.” It is a chicken-and-egg problem that results in or is caused by women receiving “less peer recognition, fewer invitations to speak at meetings, and less grant support” [ 4]. Peter Lawrence argues that the more “male” tendency to aggressiveness and self-promotion gives men a competitive edge over the average woman; one suggestion is that scientists' contributions be evaluated by actually reading the published papers and consulting experts as to their significance, rather than counting the number of articles or dwelling on the profile of the journal [ 5]. Werb argues that at least part of the solution is to make scientific life more family-friendly (e.g., by encouraging employers to make childcare available), so that having children does not pose an insurmountable obstacle to a challenging career. These measures, along with ensuring fair hiring and promotion procedures, are tasks for universities and research institutions.

Scientific organizations can also do a lot. The ASCB has been at the forefront of promoting gender equality in the molecular life sciences in the US, having established Women in Cell Biology in the 1970s. The ASCB Web site provides information and important links for all interested. In Europe, too, many resources are available from the European Molecular Biology Organization (EMBO), ELSO, the European Commission, and others. Key advice is that women scientists should obtain mentoring—and, in turn, be good mentors—and network among themselves. The Database of Expert Women in the Molecular Life Sciences is a complementary effort, undertaken by the Career Development Committee of ELSO ( http://www.elso-cdc.org), which established the criteria for inclusion. An expert woman can register if she is of European nationality or working in Europe, and she must be first or last author of at least one paper in a major international journal within the past three years. Adherence to these criteria ensures the usefulness of the database in the following ways.

First, the database will help organizers of scientific meetings identify women speakers to invite. According to Susan Forsburg, “this matters, because the exposure on the podium can significantly affect careers by exposing the speaker to potential post-docs, collaborators, job opportunities, or prizes—and, of course, further speaking opportunities” [ 6]. It has become unacceptable to organize an international meeting without a reasonable number of women on the invited speaker list (ELSO recommends a target of 35% women). Some organizations that sponsor European meetings, such as EMBO and the Federation of Biochemical Societies, stipulate in their guidelines to meeting organizers that gender balance should be considered when assembling the speaker list. Nevertheless, it is still true today that too many European meetings feature no or very few women speakers. Achieving gender balance can be a challenge; indeed, Werb notes that ASCB-sponsored meetings have had difficulty identifying and engaging appropriate women to take part [ 4]. The database can help serve this need by drawing attention to more junior women whose names may not at first spring to mind.

Second, our peer-review system, by its very name, requires that gender balance be considered when assembling commissions, grant review panels, and editorial boards, as well as ad hoc reviewers contributing to all three. Several studies, in fact, have revealed gender bias in the evaluation of proposals [ 1, 7]. But achieving gender balance is as much a challenge for grants administrators and journal editors as it is for meeting organizers.

Finally, the database allows search committees to identify qualified women in a desired field from whom applications for group leader, professor, and other positions may be solicited. Science is above all driven by excellence, and no one would select his or her next colleague solely because she is a woman. However, a viable strategy is to increase the number of female applicants for each job and then select the best person. When the proportion of women applicants increases, more women will rise to the top.

The Database of Expert Women in the Molecular Life Sciences, which was launched in September at the annual ELSO meeting, already contains more than 300 entries. The hope is that many scientists around the world will include the database among their favorite bookmarks. Seeing the women who are already experts, one can believe that the “scissors” will close.

**Box 1**

ASCB: http://www.ascb.org

ELSO Career Development Committee: http://www.elso-cdc.org

EMBO Women in Science: http://www.embo.org/gender/links.html

European Commission Science and Society: http://europa.esn.be/comm/research/science-society/home_en.cfm

## Abbreviations

ASCB: American Society for Cell Biology
ELSO: European Life Sciences Organization
EMBO: European Molecular Biology Organization
